# Photothermal Response Induced by Nanocage-Coated Artificial Extracellular Matrix Promotes Neural Stem Cell Differentiation

**DOI:** 10.3390/nano11051216

**Published:** 2021-05-04

**Authors:** Seunghyun Jung, Nathaniel Harris, Isabelle I. Niyonshuti, Samir V. Jenkins, Abdallah M. Hayar, Fumiya Watanabe, Azemat Jamshidi-Parsian, Jingyi Chen, Michael J. Borrelli, Robert J. Griffin

**Affiliations:** 1 Department of Physiology and Cell Biology, University of Arkansas for Medical Sciences, Little Rock, AR 72205, USA; shjung@uams.edu (S.J.); MjBorrelli@uams.edu (M.J.B.); 2 Department of Radiation Oncology, University of Arkansas for Medical Sciences, Little Rock, AR 72205, USA; SVJenkins@uams.edu (S.V.J.); JamshidiAzema@uams.edu (A.J.-P.); 3 Mechanical Engineering, University of Arkansas, Fayetteville, AR 72701, USA; nqharris@uark.edu; 4 Department of Chemistry and Biochemistry, University of Arkansas, Fayetteville, AR 72701, USA; iniyonsh@uark.edu (I.I.N.); chenj@uark.edu (J.C.); 5 Department of Neurobiology and Developmental Sciences, University of Arkansas for Medical Sciences, Little Rock, AR 72205, USA; AMHayar@uams.edu; 6 Center for Integrative Nanotechnology Sciences, University of Arkansas, Little Rock, AR 72204, USA; fxwatanabe@ualr.edu; 7 Department of Radiology, University of Arkansas for Medical Sciences, Little Rock, AR 72205, USA

**Keywords:** gold nanocages, artificial extracellular matrix, photothermal, neuronal differentiation, neuronal maturation

## Abstract

Strategies to increase the proportion of neural stem cells that differentiate into neurons are vital for therapy of neurodegenerative disorders. In vitro, the extracellular matrix composition and topography have been found to be important factors in stem cell differentiation. We have developed a novel artificial extracellular matrix (aECM) formed by attaching gold nanocages (AuNCs) to glass coverslips. After culturing rat neural stem cells (rNSCs) on these gold nanocage-coated surfaces (AuNC-aECMs), we observed that 44.6% of rNSCs differentiated into neurons compared to only 27.9% for cells grown on laminin-coated glass coverslips. We applied laser irradiation to the AuNC-aECMs to introduce precise amounts of photothermally induced heat shock in cells. Our results showed that laser-induced thermal stimulation of AuNC-aECMs further enhanced neuronal differentiation (56%) depending on the laser intensity used. Response to these photothermal effects increased the expression of heat shock protein 27, 70, and 90α in rNSCs. Analysis of dendritic complexity showed that this thermal stimulation promoted neuronal maturation by increasing dendrite length as thermal dose was increased. In addition, we found that cells growing on AuNC-aECMs post laser irradiation exhibited action potentials and increased the expression of voltage-gated Na+ channels compared to laminin-coated glass coverslips. These results indicate that the photothermal response induced in cells growing on AuNC-aECMs can be used to produce large quantities of functional neurons, with improved electrochemical properties, that can potentially be transplanted into a damaged central nervous system to provide replacement neurons and restore lost function.

## 1. Introduction

During the development of the mammalian central nervous system (CNS), neural stem cells (NSCs) differentiate into neurons and glial cells. Glial cells, consisting of astrocyte, microglia, and oligodendrocyte cells constitute a large fraction of the mammalian brain. Astrocytes play key roles in numerous functions within CNS, including glutamate, ion, and water homeostasis, defense against oxidative stress, energy storage, scar formation, and tissue repair [1,2,3,4]. After CNS injury, astrocytes communicate with surrounding vascular systems, resulting in the removal of protein aggregates, such as β-amyloid and α-synuclein [5,6,7]. Microglia are the phagocytic cells of the nervous system and are involved in synaptic organization and trophic neuronal support during development [8,9]. Oligodendrocytes assemble myelin, a multilayered sheath of membrane that spirally wraps around axonal segments to enable fast impulse propagation [10,11,12]. In contrast, neurons are the basic working unit of the brain that transmits information to other nerve, muscle, and gland cells. The fundamental process of synaptic transmission is the action potential, which is a propagating electrical signal that is a shift in the neuron’s electrical potential caused by the flow of ions across the neural membrane. Brain injury often causes the death of neurons and glial cells, which can destroy intricate neuronal circuitry. The current paradigm is that the adult brain has a limited capacity for injury repair due to an inadequate capability to replace damaged neurons. Hence, the ability to up-regulate neuronal renewal is a promising therapeutic strategy to replace lost neurons and restore lost functions that stem from neurodegenerative disease processes. However, the differentiation mechanisms of neural stem cells into specific cell lineages are complicated and mostly unknown. Multiple aspects of the microenvironment, e.g., extracellular signals (growth factors and oxygen tension), the extracellular matrix (ECM), and intercellular signaling (cytokines, exosomes, and cell polarity) influence the differentiation of neural stem cells into specific lineages [13]. During in vitro culture, three-dimensional structures, such as grid patterns of extracellular matrix protein, increase NSC differentiation into neurons while decreasing differentiation into glial cells [14]. Recent stem cell studies demonstrated that NSCs respond strongly to the nanoscale topography of the matrix environment such as nanofiber and 3D-printed structures [15,16,17,18,19]. However, many previous studies have used limited types of nanoscopic geometries (e.g., nano-wires and nano-needles) as optimal artificial extracellular matrices (aECMs) [20], which limits control over differentiation into specific cell types. Therefore, we have studied substrates containing multiple types of gold nanomaterials (gold nanorods and gold nanocages) to function as aECMs in an attempt to improve directing differentiation of NSCs into neurons.

Gold nanoparticles (AuNPs) were used because of their known unique properties, including biocompatibility, chemical and physical stability, efficient thermal transduction, optical properties, and facile surface functionalization [21,22,23]. When the AuNPs are exposed to light, the electrons move away from their equilibrium position, creating a resonant coherent oscillation called the localized surface plasmon resonance (LSPR), which induces a strong absorption of the incident light [24]. The AuNPs release the absorbed energy as heat, making AuNPs potent agents for photothermal therapy. The Xia’s group studied photothermal conversion efficiencies with varying types of Au nanostructures (Au nanocages, Au nanohexapods, and Au nanorods). The photothermal conversion efficiencies were found to be different depending on nanoparticle morphology and shape [25]. By controlling the edge length, wall thickness, or the length of the arms of nanoparticles, the localized surface plasmon resonance peak position could be tuned, which influences absorption and conversion of near-infrared light into heat. Thus, control over the absorption and scattering of light plays a key role in the efficiency of light-to-heat conversion of nanoparticles [26,27]. In the specific case of the nanocage structure used here for the majority of our work, due to the unique size and structure of AuNCs, we and others have found that the total light extinction of AuNCs is dominated by absorption, resulting in high efficiency of light-to-heat conversion, which is likely due to the thin walls and hollow nature of the AuNC [28,29]. Recently, stimulation methods have been studied and reported to either excite or inhibit neuronal activities via AuNPs-mediated laser stimulation [30]. However, in this study, AuNPs were added to cell cultures and laser irradiation was applied. Due to the inability to control the precise number of AuNPs within or in contact with cells, the resultant photothermal effect could not be calculated to determine the accurate thermal dose that triggered desirable cellular responses.

We have developed a novel nanomaterial validation platform composed of tissue culture surfaces coated with an array of different nanoparticles. We previously reported this unique method by adhering 50 × 50 nm hollow, cubic, gold nanocages (AuNCs) onto a glass coverslip at varying densities, to induce, characterize, and apply precise thermal doses to cancer cells growing on these surfaces [31]. In the current study, we found that the AuNC-aECMs promoted the differentiation of rNSCs into neurons to a moderate extent. To induce a more robust differentiation response of rNSCs, laser irradiation was applied to the cells growing on AuNC-aECMs to introduce precise amounts of photothermal exposure in the adherent cells. After this intervention, we observed increased neuronal differentiation and maturation compared to either laser alone or cells grown on AuNC-ECMs alone. The ability of photothermal treatment to induce changes in cell morphology and protein expressions that determine the specific differentiation cascade and potential for biomedical applications is discussed in the context of our results.

## 2. Materials and Methods

### 2.1. Materials and Chemicals

Sodium carbonate (Na_2_CO_3_), sodium bicarbonate (NaHCO_3_), methanol (MeOH), ethanol (EtOH), sulfuric acid (H_2_SO_4_), nitric acid (HNO_3_), hydrochloric acid (HCl), 30% hydrogen peroxide (H_2_O_2_), and 3-mercaptopropyltrismethoxysilane (MPTMS) were purchased from Sigma Aldrich (St. Louis, MO, USA). All experiments were performed using 18 MΩ H_2_O unless specified otherwise.

### 2.2. Gold Nanocage (AuNC) Preparation

AuNCs were synthesized using the galvanic replacement reaction between the silver nanocubes and tetrachloroauric acid in aqueous solution modified from previous publications [32,33].

### 2.3. Gold Nanocage-Artificial Extracellular Matrices (AuNC-aECMs) Preparation

Glass coverslips (circular, 12.5 mm diameter, Fisher scientific, Cat#12-545-81P) were treated with aqua regia (3:1 HCl:HNO_3_) for 60 min at room temperature and then with piranha solution (3:1 H_2_SO_4_:H_2_O_2_) at 60–65 °C for 40 min. Afterwards, the coverslips were rinsed with 18 MΩ water (MilliporeSigma, Burlington, MA, USA), followed by rinses twice with 100% MeOH. Following the last MeOH rinse, coverslips were treated with 10% (trismethoxy)-mercaptopropyl silane (MPTMS: Sigma Aldrich, St. Louis, MO, USA, Cat#175617) in MeOH for 65–72 h at 37 °C. Then, glass coverslips were rinsed thrice with MeOH, placed into separate wells in a 24-well plate containing 1 mL of 100% MeOH per well, which was then floated atop the water surface of an ultrasonic cleaning bath (Fisher Scientific, Waltham, MA, USA, Cat# 15337410) and sonicated for 5 min. Following two final rinses with 100% MeOH, the coverslips were placed, silanized face up, onto a hot plate for 5 min at 100 °C. Next, the silanized coverslips were placed into a new 24-well plate containing sodium carbonate-bicarbonate buffer (pH = 9.1, 5 mM) into which AuNCs (12.8 fmol) were added and the plates were incubated for 24 h at 37 °C. The AuNC-aECMs were rinsed twice with distilled water and allowed to dry in the plate. AuNC-aECMs were incubated with 70% EtOH for 30 min at room temperature to sterilize them. After the sterilizing procedure, the AuNC-aECMs were rinsed twice with sterilized 18 MΩ water.

### 2.4. AuNC-aECM Characterization

AuNC-aECMs were mounted onto 12.2 mm dia. × 10 mm high aluminum disks (Ted Pella, Inc. Redding, CA, USA) using double-sided carbon tapes, and their surfaces were covered by a ≈3 nm thick carbon films (Gatan 681 Ion Beam, Gatan, Inc. Pleasanton, CA, USA). The carbon films were deposited. Then, AuNC-aECMs were mounted on a stainless steel specimen holder and their edges were grounded to the holder by a conductive copper tape prior to being inserted into a JEOL JSM-7000F (JEOL USA, Peabody, MA, USA) field emission scanning electron microscope. A series of secondary electron images with varying magnifications on each specimen were obtained at specimen bias ranging from 5 to 15 kV.

### 2.5. Laminin Coating

The glass coverslips and AuNC-aECMs were coated with 0.1 mg/mL of poly-D-lysine (Sigma Aldrich, St. Louis, MO, USA, Cat# 6407) for 10 min at room temperature, and then surfaces were rinsed twice with distilled water for 5 min. The surfaces were treated with 2 μg/cm^2^ of laminin (Sigma Aldrich, St. Louis, MO, USA, Cat# L2020) for 2 h in an incubator (37 °C) and then rinsed twice with PBS.

### 2.6. Cell Culture and rNSC Differentiation

Rat fetal neural stem cells (rNSCs) were purchased from GIBCO (Waltham, MA, USA, Cat# N7744200-kit) and were seeded at a density of 37,500 cells/cm^2^ onto laminin-coated glass coverslips or laminin-coated AuNC-aECMs. The rNSCs were cultured in StemPro NSC complete Medium (GIBCO-kit) in an incubator (5% CO_2_ at 37 °C). After 2 days, the medium was switched to Neuronal Differentiation Medium (GIBCO-kit), and the medium was replaced every 3 days with neuronal differentiation medium.

### 2.7. Photothermal Treatment

The laminin-coated AuNC-aECMs were placed into a 24-well plate, and rNSCs were seeded at a density of 37,500 cells/cm^2^; then, they were incubated with 5% CO_2_ at 37 °C. After 2 days, the wells were covered with 1 mL of StemPro NSC complete Medium. The laser spot size was adjusted to 12.5 mm diameter, and laser irradiation was made using a Diomed 25 laser at 808 nm with a fluence of 0.5–5.0 W/cm^2^ for 1–5 min. The cells were allowed to equilibrate to room temperature for 3–5 min prior to laser application. Afterwards, cells were returned to the incubator, and the medium was switched from StemPro NSC complete Medium to Neuronal Differentiation Medium 24 h later. The medium was replaced every 3 days with Neuronal Differentiation Medium prior to measurement of neuronal differentiation and patch-clamp recording. To quantify the effects of photothermal response of laminin-coated AuNC-aECMs, the temperature of surrounding medium of laminin-coated AuNC-aECMs was measured with a thermocouple inserted into the well and placed close to the surface of the coverslip post laser treatment.

### 2.8. Measurement of Cell Viability

rNSCs were seeded onto AuNC-aECMs at a density of 37,500 cells/cm^2^ and were cultured with StemPro NSC complete medium. The medium was replaced every 3 days with StemPro NSC complete medium. After 4 days, laser irradiation was applied using a Diomed 25 at 808 nm (0.5–5.0 W/cm^2^ for 1–5 min). After 24 h, the cells were collected into 15 mL tubes by using a StemPro Accutase Cell Dissociation Reagent (GIBCO, Waltham, MA, USA, Cat#A1110501). The cells were rinsed with PBS and fixed for 15 min using 4% paraformaldehyde with 3% BSA in 0.1% of Triton-X-100 solution in PBS. The cells were incubated with Fixable Viability Dye eFluor 780 (FVD 780; Invitrogen, Carlsbad, CA, USA, Cat# 65-0865) for 30 min at 4 °C. The cells were rinsed with flow cytometry buffer (2% BSA in PBS) and cell viability was assessed via flow cytometry.

### 2.9. Immunocytochemistry

AuNC-aECMs were rinsed with PBS and fixed for 15 min using 4% paraformaldehyde. AuNC-aECMs were then, treated with a blocking solution with 3% BSA in 0.1% of Triton-X-100 solution in PBS for an hour. The AuNC-aECMs were incubated for 24 h at 4 °C with the following antibodies: β-III-tubulin (1:500, Abcam, Cambridge, UK, Cat# AB78078), GFAP (1:1000, Dako, Santa Clara, CA, USA, Cat# Z0334), MAP2 (1:250, Sigma Aldrich, St. Louis, MI, USA, Cat# M4403), and Nav1.6/SCN8A (1:250, Alomone Labs, Jerusalem, Israel, Cat# ASC-009). Afterwards, cells were rinsed thrice with 0.1% of Triton-X-100 solution in PBS. The cells were incubated for 2 h at room temperature with the following secondary antibodies: Goat anti-mouse IgG (H+L) Alexa-488 (1:500, Molecular Probes, Eugene, OR, USA, #A11001), Goat anti-rabbit IgG (H+L) Alexa-594 (1:500, Molecular Probes, Eugene, OR, USA, Cat# A11008) followed by a 10 min incubation with Hoechst 33342 (1 µg/mL) at room temperature. The cells were rinsed thrice with 0.1% of Triton-X-100 solution in PBS and rinsed with PBS.

### 2.10. Assessment of Dendrite Length and Number Using the Neurite Tracing Program

A neurite tracing algorithm was developed in MATLAB to assess trends in neurite length due to substrate type and neuron location. Similar to other programs that are available currently for tracing neurons, such as Neurolucida (MBF Bioscience, Williston, VT, USA), the algorithm receives a series of inputs from the user to trace the perimeter of each soma, the neurites branching from the soma, and neurites branching from previously traced neurites. Visible connections between neurons are indicated. The user defines the value of dark pixels on the substrate to automatically calculate neurite diameters, if desired. A Graphical User Interface (GUI) allows the user to navigate around the image, zoom in and out, and save snapshots of the tracing process. The neurite length, branching directions, branch number, and the number of connections between each neuron is saved into an excel spreadsheet for later use. Especially relevant to the current study, the neurite lengths were calculated by adding up the distances between each neurite point traced by the user. Neurites that are connected by the user are counted as originating from both neurons extending in symmetrically opposite directions.

### 2.11. Patch-Clamp Recording

The in vitro electrophysiological setup for patch clamp recordings included an anti-vibration table (TMC, Peabody, MA, USA), an Olympus BX51WI microscope equipped with infrared DIC and epifluorescence, and a fine motorized micromanipulator (model# MX7500, Siskiyou Corp., Grants Pass, OR, USA, 4 axes: X, Y, Z and oblique, each has a moving resolution of 0.1 µm, and a traveling distance of 2 cm) [34,35]. The micromanipulators were installed near a custom-made patch-clamp recording chamber heated to 30 °C using a 12 V direct current (DC) battery with temperature controlled by a Bipolar Temperature Controller (TC2BIP, Cell MicroControls, Norfolk, VA, USA). The motorized micromanipulator holds the headstage of the patch clamp amplifier (Multiclamp 700B, Molecular Devices, Sunnyvale, CA, USA). For recording, a single coverslip was placed in a recording chamber and continuously perfused at the rate of 1.5 mL/min with artificial cerebrospinal fluid (aCSF) equilibrated with 95% O_2_–5% CO_2_ (composition in mM: 124 NaCl, 26 NaHCO_3_, 3 KCl, 2 MgCl_2_, 2 CaCl_2_, 0.4 ascorbic acid, 2 sodium pyruvate, and 20 glucose). During electrophysiological recordings, the preparation was viewed with the 40× objective of an Olympus BX51WI microscope equipped with epifluorescence. Using a remote control, the optical zoom of the camcorder was increased to the maximum (image width ≈ 100 µm) when recording from a single neuron and it was reduced as needed (image width ≈ 500 µm) to identify the location of the neuron. The camcorder was also used to take photos (still images), which were taken while the object was slowly moved up and down to bring in focus the entire 3D morphological structure of the neurons that were labeled with fluorescent dyes. Recording pipettes were pulled from borosilicate glass capillaries with an inner filament (1.5 mm o.d., World Precision Instruments, Sarasota, FL, USA) on a pipette puller (P-97, Sutter Instrument Company, Novato, CA, USA). Recordings were made using a Multiclamp-700B amplifier, and analog signals were low-pass filtered at 2 kHz and digitized at 5 kHz using a Digidata-1440A interface and pClamp10 software (Molecular Devices, Sunnyvale, CA, USA). For whole-cell patch clamp recordings, patch pipettes (−8–12 MΩ) were filled with a solution of the following composition, (in mM) 125 potassium gluconate, 1 NaCl, 10 phosphocreatine ditris salt, 4 MgATP, 0.3 GTP, 0.5 EGTA, and 10 HEPES (pH 7.3 with NaOH, osmolarity 290 mOsm). The junction potential was 9–10 mV, and all reported voltage measurements were uncorrected for these potentials. Only neurons with access resistance <30 MΩ were included in this study. No series resistance compensation was performed. In all experiments, 0.02% Lucifer Yellow (Molecular Probes, Eugene, OR, USA) was added to the intracellular solution for in situ labeling.

### 2.12. Measurement of Heat Shock Protein 27, 70, and 90α Expression

rNSCs were seeded onto laminin-coated AuNC-aECMs at a density of 37,500 cells/cm^2^ and cultured with StemPro NSC complete medium. The medium was replaced every 3 days with StemPro NSC complete medium. After 4 days, laser application was applied using a Diomed 25 at 808 nm (2.0 W/cm^2^ for 1 min). The cells were collected at varying time points (0 h–24 h) and rinsed with PBS. The cells were fixed for 15 min using 4% paraformaldehyde with 3% BSA in 0.1% of Triton-X-100 solution in PBS, treated with a permeabilization solution with 90% MeOH in PBS, and then incubated for 10 min on ice. The cells were rinsed with PBS twice and incubated for 1 h at room temperature with following antibodies: Hsp27 antibody (1:500, Santa Cruz Biotechnology, Dallas, TX, USA, Cat# SC-1094), Hsp70 antibody (1:500, Santa Cruz Biotechnology, Dallas, TX, USA, Cat# SC-24), and Hsp90α (1:500, Santa Cruz Biotechnology, Dallas, TX, USA, Cat# SC-8262). The cells were rinsed with PBS twice and incubated for 30 min at room temperature with the following secondary antibodies: goat anti-mouse IgG (H+L) Alexa-488 (1:500, Molecular Probes, Eugene, OR, USA) and donkey anti-goat IgG (H+L) Alexa-555 (1:500, Molecular Probes, Eugene, OR, USA). The cells were rinsed with PBS twice and resuspended with flow cytometry buffer (2% BSA in PBS). The heat shock protein 27, 70, and 90α expression levels were measured using flow cytometry. For bulk heating experiments, the laminin-coated glass coverslips were placed into a 24-well plate and rNSCs were seeded at a density of 37,500 cells/cm^2^. After 4 days, plates were sealed with parafilm, enclosed in a plastic bags, and then submerged in a circulating water bath (Thermo Fisher Scientific, Waltham, MA, USA, Thermo DC10) at 43 °C for 30 min. Then, plates were returned to the incubator (5% CO_2_ at 37 °C), and after varying incubation time points, the cells were collected and the heat shock protein 27, 70, and 90α expression levels were measured by flow cytometry.

### 2.13. Statistical Analysis

The results are expressed as the mean ±S.D. of triplicate experiments. Statistical significance was evaluated using a one-way ANOVA. Data are presented as the means ±SD of three independent experiments. * *p* < 0.05 vs. laminin-coated AuNC-aECM in the absence of laser irradiation group.

## 3. Results

### 3.1. Characterization of the AuNC-aECMs and Laminin-Coated AuNC-aECMs

**Figure 1:**
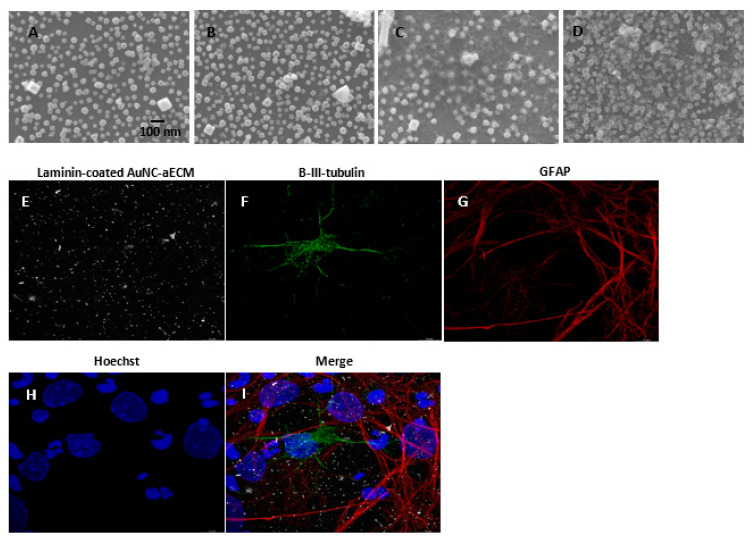
Characterization of AuNC-aECMs and laminin-coated AuNC-aECMs. (**A**–**D**) Scanning electron microscopy of AuNC-aECMs. The quantity of AuNCs can be controlled by reaction buffer solution and the concentration of AuNC colloidal solution (**A**: 12.8 fmol, 32 AuNCs/μm^2^ and **B**: 25.6 fmol, 54 AuNCs/μm^2^). The AuNC-aECMs were coated with laminin (**C**: 32 AuNCs/μm^2^ and **D**: 54 AuNCs/μm^2^). The cells growing on laminin-coated AuNC-aECMs (32 AuNCs/μm^2^) were analyzed with immunostaining assay and fluorescent images were obtained using Leica—Lightning Light Laser Confocal Microscope SP8X (**E**–**I**). (**E**) Laminin-coated AuNC-aECM, (**F**) β-III-tubulin immunostaining (neuron marker), (**G**) Glial fibrillary acidic protein (GFAP) immunostaining (astrocyte marker), (**H**) Hoechst staining, (**I**) Merged image.

AuNCs were coated onto glass coverslips as reported previously [31]. AuNC-aECMs were characterized using scanning electron microscopy (SEM) to validate that the density and spacing of the AuNCs could be controlled and the general features of the coated surface were consistent across batches (Figure 1A,B). rNSCs seeded onto AuNC-aECMs did not attach to the surface and then died within 5 days post seeding. Therefore, we coated AuNC-aECMs with laminin to mimic natural ECMs and provide an optimal environment for rNSCs to attach to AuNC-aECMs and grow. To identify morphological changes of AuNC-aECMs post laminin coating, laminin-coated AuNC-aECMs were characterized by SEM (Figure 1C,D), and the SEM images showed that AuNCs that were deposited onto the glass coverslips retained their unique nanocage structures, and varying the initial AuNC quantity from 12.8 fmol and 25.6 fmol AuNC yielded coverage of 32 and 54 AuNCs/μm^2^, respectively. rNSCs were seeded onto laminin-coated AuNC-aECMs (12.8 fmol, 32 AuNCs/μm^2^) and subsequently analyzed with immunostaining to identify whether rNSCs underwent differentiation into varying cell lineages such as neurons and astrocytes (Figure 1E–I). The results showed that rNSCs differentiated into neurons and astrocytes by contact with the laminin-coated AuNCs onto glass coverslips.

### 3.2. Effects of the Varying Coverage Densities of AuNCs onto Laminin-Coated AuNC-aECMs and Photothermal Response of Laminin-Coated AuNC-aECMs on Neuronal Differentiation Capacity in Rat Neural Stem Cells

**Figure 2:**
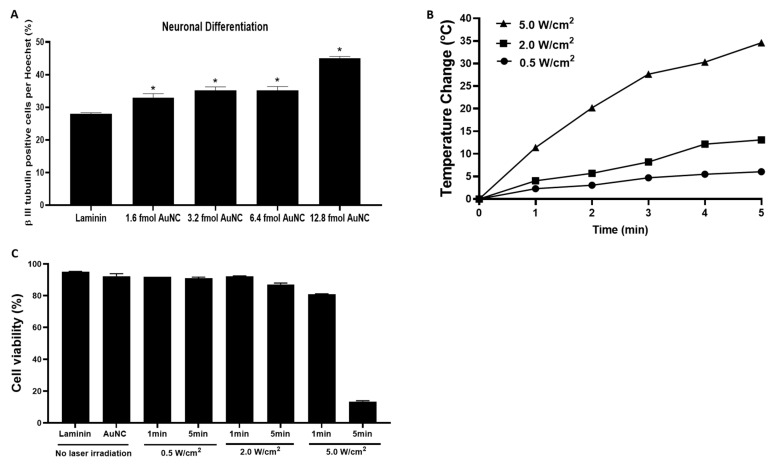
Cell viability and neuronal differentiation of rNSCs grown on laminin-coated AuNC-aECMs with/without laser irradiation. rNSCs were seeded onto laminin-coated AuNC-aECMs with varying coverage densities of AuNCs. Cells were analyzed with immunocytochemistry at 16 days post seeding (**A**). The laminin-coated AuNC-aECMs were treated by laser irradiation with varying parameters (0.5–5.0 W/cm^2^ for 1–5 min), and the temperature at the surface in wells containing 1 mL cell culture medium was measured by a thermocouple (**B**). rNSCs were seeded onto laminin-coated AuNC-aECMs, and after 4 days, the cells were treated with indicated laser irradiation (0.5–5.0 W/cm^2^ for 1–5 min). After 24 h, the cells were collected, and then, cell viability assay was performed using flow cytometry (**C**). Values represent the means ± SDs of three independent experiments. * *p* values < 0.05 vs. the control group (laminin-coated glass coverslip).

We investigated whether the manipulation of laminin-coated AuNC-aECMs could specifically promote the differentiation of rNSCs into neurons. AuNC-aECMs with varying coverage densities of AuNCs were produced, and rNSCs were seeded onto these surfaces. After 16 days in culture, immunocytochemistry was performed to quantify the neuronal populations (Figure 2A). We found that laminin-coated AuNC-aECMs significantly increased the differentiation of rNSCs into neurons in a coverage density-dependent manner compared to laminin-coated glass coverslip cultures, with maximal neuronal differentiation observed in 12.8 fmol (32 AuNC/cm^2^) of laminin-coated AuNC-aECMs. However, after seeding rNSCs onto 25.6 fmol (54 AuNC/cm^2^) of laminin-coated AuNC-aECMs, rNSCs did not attach to the surfaces and eventually died within 5–7 days. Therefore, we used 12.8 fmol (32 AuNC/cm^2^) of laminin-coated AuNC-aECMs for further experiments as the standard surfaces.

In attempting to further enhance neuronal differentiation, we hypothesized that laser-induced thermal stimulation of laminin-coated AuNC-aECMs might induce NSC plasma membrane heat stress that subsequently influences the differentiation of NSCs into neurons. To determine the laser-induced thermal dose delivered to laminin-coated AuNC-aECMs, post laser irradiation, the temperature of the surrounding medium of laminin-coated AuNC-aECMs (12.8 fmol; 32 AuNCs/μm^2^) was measured (Figure 2B). The results showed that laminin-coated AuNC-aECMs increased the temperature of surrounding medium in both a laser intensity-dependent and laser exposure duration-dependent manner. In our recent report [31], we observed AuNCs onto the surfaces did not show significant morphological changes post laser irradiation. However, it should be remembered that any changes in nanoparticle composition, size, and shape will affect the light-to-heat conversion ability of nanoparticles, which would have a profound impact on photothermal responses of nanoparticles upon repeated laser exposures [36]. To characterize the absorption and scattering contribution of nanoparticles, employing new approaches described for diffuse-reflectance UV/vis/NIR spectroscopy could be a potent analytical technique [37]. To determine the appropriate parameters of laser irradiation that did not alter cell viability, we performed the cell viability assay using flow cytometry (Figure 2C). Following laser irradiation with varying laser intensity-duration combinations (0.5–5.0 W/cm^2^ for 1–5 min), cellular toxicities were not observed up to 2.0 W/cm^2^ for 1 min. Cell viability decreased slightly at 2.0 W/cm^2^ for 5 min (87.2% ± 0.6) and 5.0 W/cm^2^ for 1 min (81.1% ± 0.1) compared to the absence of laser irradiation (95.1% ± 0.3). The highest laser exposure of 5.0 W/cm^2^ for 5 min induced significant rNSCs cytotoxicity (13.6% ± 0.4).

### 3.3. Effects of Photothermal Response of Laminin-Coated AuNC-aECMs on Neuronal Differentiation Capacity in Rat Neural Stem Cells

**Figure 3:**
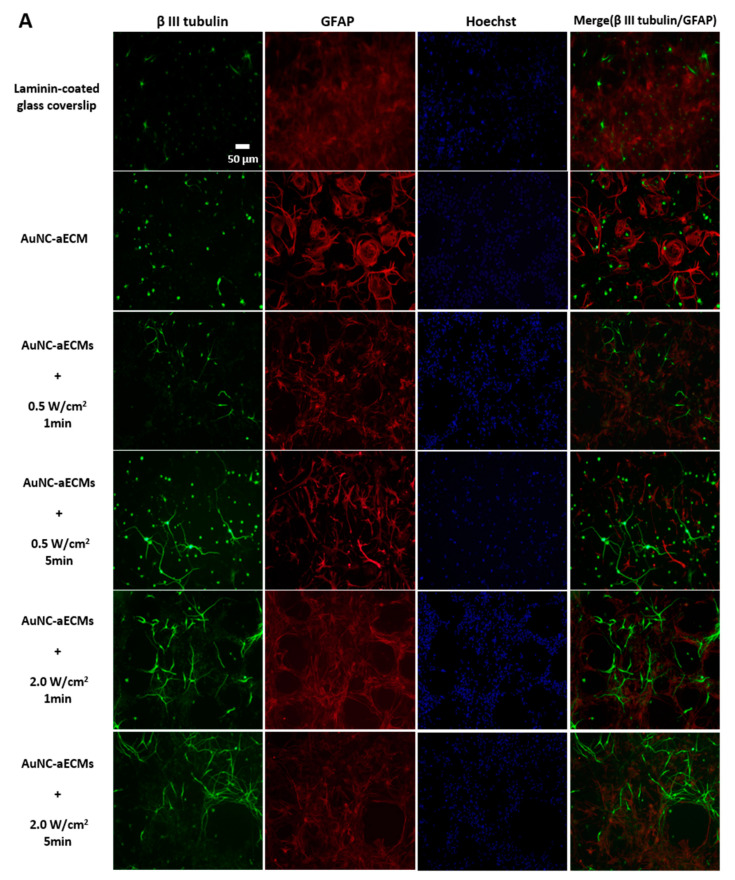

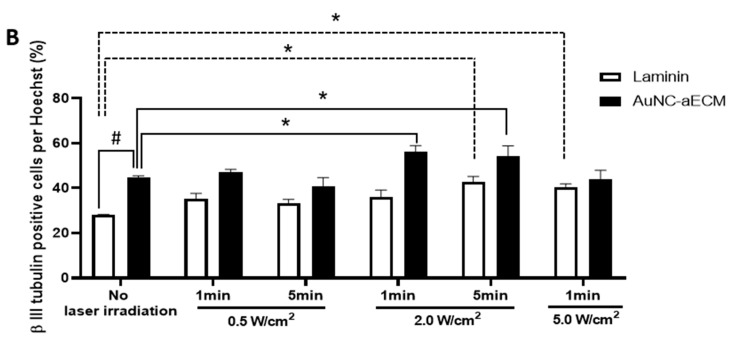
Neuronal differentiation of rNSCs grown on laminin-coated glass coverslips and AuNC-aECMs with/without laser irradiation. rNSCs were seeded onto both laminin-coated glass coverslips and laminin-coated AuNC-aECMs and after 2 days, cells were treated with indicated laser irradiation parameters (0.5–5.0 W/cm^2^ for 1–5 min). After 14 days, cells were analyzed with immunostaining assay (**A**,**B**). Differentiated neuron and astrocyte cell populations were identified with β-III-tubulin (neuron marker) and GFAP (astrocyte marker). Values represent the means ± SDs of three independent experiments. # *p* values < 0.05 vs. the control group (laminin-coated glass coverslip). * *p* < 0.05 vs. laminin-coated AuNC-aECM in the absence of laser irradiation group.

rNSCs were seeded onto both laminin-coated glass coverslips and laminin-coated AuNC-aECMs and then treated with laser irradiation to determine if the photothermal response of laminin-coated AuNC-aECMs promoted additional neuronal differentiation. Immunostaining was performed to quantify differentiated neuron and astrocyte populations (Figure 3A). Figure 3A illustrates that increased β-III-tubulin positive cells were identified in laminin-coated AuNC-aECMs and laminin-coated AuNC-aECMs post laser irradiation compared to only laminin-coated glass coverslips. Furthermore, we found that as laser intensity was increased, β-III-tubulin positive cells had more mature morphology indicated by long dendrites and fine neurite structures.

Figure 3B shows that laminin-coated AuNC-aECMs without laser irradiation increased neuronal differentiation markedly (44.6% ± 0.7) compared to laminin-coated coverslips (27.9% ± 0.3). In laminin-coated AuNC-aECMs with laser irradiation, we found no significant differences in neuronal differentiation except for 2.0 W/cm^2^ for 1 min (56% ± 2.9) and 2.0 W/cm^2^ for 5 min (54.1% ± 4.7) compared to laminin-coated AuNC-aECMs cultures in the absence of laser irradiation (44.6% ± 0.7). However, slightly increased neuronal differentiation was found in 0.5 W/cm^2^ for 1 min (47.2% ± 0.9) and 0.5 W/cm^2^ for 5 min (46% ± 2.4) compared to the absence of laser irradiation. On laminin-coated glass coverslips, we found significantly increased neuronal differentiation following laser exposure of 2.0 W/cm^2^ for 5 min (42.7% ± 1.8) and 5.0 W/cm^2^ for 1 min (40.6% ± 0.9) compared to laminin-coated glass coverslips without laser irradiation (27.9% ± 0.3). These results showed that laser irradiation alone increased neuronal differentiation regardless of the presence of AuNCs, although laser-induced thermal dose produced by laminin-coated AuNC-aECMs had a greater impact on neuronal differentiation.

### 3.4. Effects of Photothermal Response of Laminin-Coated AuNC-aECMs on Morphological Changes of Neuronal Maturation in Rat Neural Stem Cells

**Figure 4:**
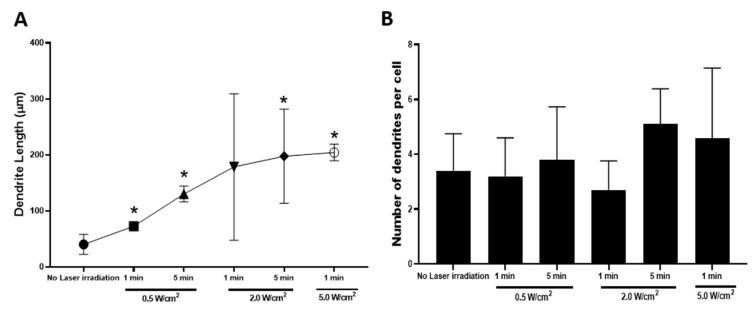
Assessment of the number and length of dendrites in differentiated neurons. rNSCs were seeded onto laminin-coated AuNC-aECMs, and after 2 days, cells were treated with indicated laser irradiation parameters (0.5–5.0 W/cm^2^ for 1–5 min). After 14 days, cells were analyzed with immunocytochemistry. The immunostaining images were analyzed with a custom-made MATLAB script to quantify the length and number of neuronal dendrites (**A**,**B**). Values represent the means ± SDs of three independent experiments. * *p* < 0.05 vs. laminin-coated AuNC-aECM in the absence of laser irradiation group.

Neuronal dendritic complexity, post laser irradiation was quantified to investigate whether the photothermal response of laminin-coated AuNC-aECMs promoted neuronal maturation. Therefore, we developed a custom-made MATLAB script to assess neuronal morphology changes to proximal, intermediate, and terminal dendrites. We applied this program to quantify the number and length of neuronal dendrites after rNSCs were exposed to laser-induced thermal stimulation of laminin-coated AuNC-aECMs (Figure 4A,B). As shown in Figure 4A, the photothermal response of laminin-coated AuNC-aECMs significantly increased the length of neuronal dendrites in both a laser intensity- and laser exposure-dependent manner. In contrast, there was no significant change in the number of neuronal dendrites in response to laser irradiation (Figure 4B). We observed a wide range of dendrite length at 2.0 W/cm^2^ for 1 and 5 min among laser irradiation groups in Figure 4A. According to immunostaining images, differentiated neurons at 2.0 W/cm^2^ for 1 and 5 min exhibited asymmetric dendrite length.

### 3.5. Effects of Photothermal Response of Laminin-Coated AuNC-aECMs on Neuronal Electrophysiological Activity in Differentiated Neurons

**Figure 5:**
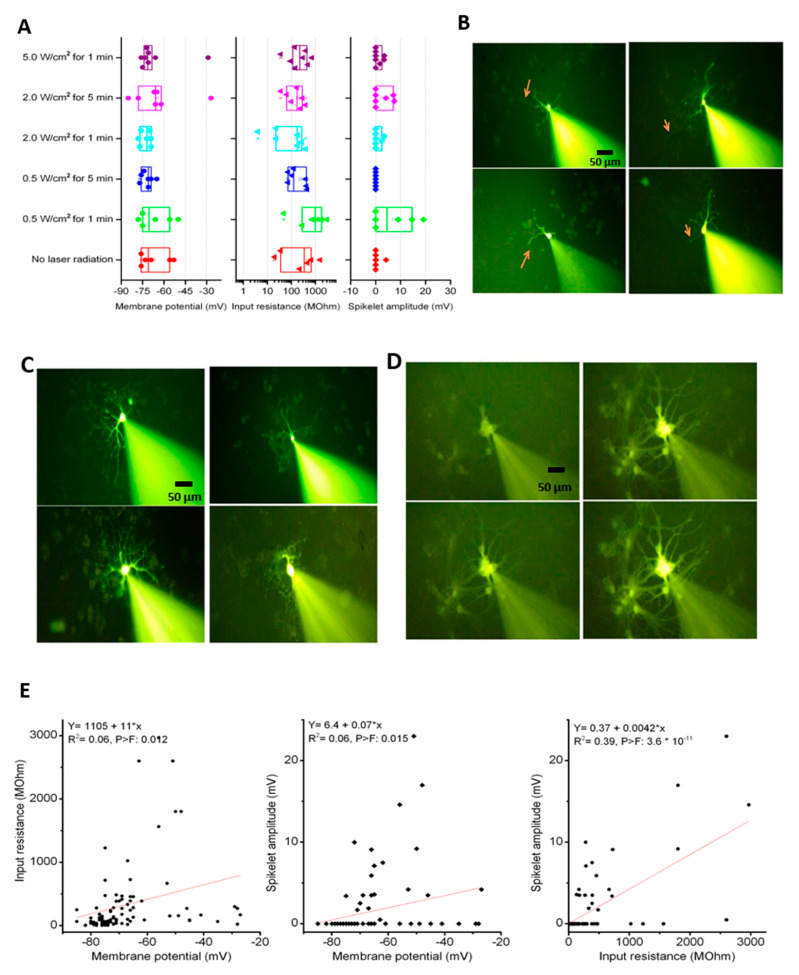

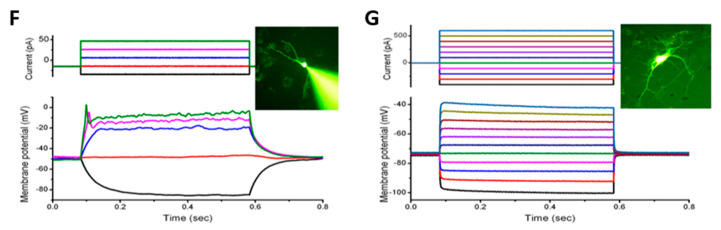
Assessment of neuronal electrophysiological activity in differentiated neurons. rNSCs were seeded onto laminin-coated AuNC-aECMs, and after 2 days, cells were treated with indicated laser irradiation parameters (0.5–5.0 W/cm^2^ for 1–5 min). After 14–16 days, cells were analyzed with the patch-clamp recordings (**A**–**G**). The resting membrane, input resistance, and spike amplitude were plotted for each coverslip (**A**). Cells that were filled with the Lucifer Yellow dye indicated morphological features post the patch-clamp recordings (**B**–**D**). (**B**) showed cells having a simple morphological structure with one identifiable fine axon (red arrow). (**C**) showed single cells having extensive dendrites in all direction. (**D**) showed that cells were connected to adjacent cells and a time-dependent filling of the neighboring cell. Correlation between electrophysiological parameters and representative electrophysiological data (**E**). A linear regression fit was performed among the different electrophysiological parameters: membrane potential, input resistance and spikelet amplitude. Note that a value of zero for the spikelet amplitude indicates that no spikelet could be evoked by member depolarization using positive current injection. (**F** and **G**) are representative voltage traces from an excitable cell (**F**) and a non-excitable cell (**G**) in response to injecting of square positive current pulses of varying amplitudes. Prob > F is a *p*-value for F-test, which is a probability with a value ranging from 0 to 1.

To identify whether the differentiated neurons growing on laminin-coated AuNC-aECMs post laser irradiation exhibit neuronal action potentials consistent with functional neurons, conventional electrophysiological measurements with patch-clamp recordings were performed (Figure 5A–G). rNSCs grown on laminin-coated AuNC-aECMs were treated with laser irradiation (0.5–5.0 W/cm^2^ for 1–5 min). Six to 12 cells were recorded using the patch clamp technique in whole-cell configuration per each laser irradiation parameter. We recorded from two control coverslips that were not subjected to laser irradiation and nine other coverslips that were laser irradiated using different stimulation intensity (0.5–5.0 W/cm^2^) and different stimulation time (1–5 min). Cells growing on laminin-coated glass coverslips did not exhibit neuronal action potentials 16 days post seeding (data not shown). However, as shown in Figure 5A, we observed that cells growing on AuNC-aECMs exhibited spikelets in the absence of laser irradiation and after laser irradiation with 0.5 W/cm^2^ for 1 min (day 17 post seeding), 2.0 W/cm^2^ for 1 min (day 18 post seeding), and 2.0 W/cm^2^ for 5 min (day 18 post seeding). A total of 20 out of 90 recorded cells exhibited spikelets to varying degrees (i.e., different amplitudes ranging from 0.5 to 23 mV; mean = 7.3 ± 1.4 n = 20). Spikelets were all-or-none miniature action potentials that were evoked in response to intracellular injection of positive current and have a threshold of −17 ± 1.6 mV (n = 20), and they indicate the beginning of expression of Na+ channels that are exclusively expressed in neurons. In contrast, cells that did not exhibit spikelets were either neurons that are not yet completely mature or non-excitable cells and members of other cell lineages (i.e., astrocytes, microglia, and oligodendrocyte cells). Despite varying laser intensity-duration combinations, we found no significant differences in the electrophysiological parameters (resting membrane potential, input resistance, and spikelet amplitude) among cells recorded in the 11 coverslips using one-way ANOVA for repeated measures. It is possible that under the same treatment conditions, randomly sampled cells can still express a wide range of electrophysiological properties. Therefore, detecting a difference might require recording from a large number of neurons, and this could not be achieved because of the limited number of cells (6–12) that could practically be recorded from each preparation.

In Figure 5B–D, these images showed morphological features of recorded cells using the Lucifer Yellow dye. As shown in Figure 5B,C, differentiated neurons that exhibit spikelets indicated simple morphological structure of axons and dendrites. Most of the differentiated neurons exhibiting spikelets had a soma with simple morphological structure consisting of two to three fine dendrites (Figure 5B). In addition, another simple morphological structure of differentiated neurons was identified with extensive neurites in all directions (Figure 5C). Additionally, in 15 of 90 (17%) recordings, a cluster of cells were labeled instead of single cell, indicating that the dye has diffused from the recorded cell to adjacent cells probably via gap junctions (Figure 5D).

In Figure 5E, we found a significant correlation among three electrophysiological parameters. In particular, we found that when the input resistance is relatively high (indicative of a smaller cell), there is a higher chance of detecting a spikelet (i.e., amplitude > zero mV). In addition, the results showed voltage traces from an excitable cell (Figure 5F) and a non-excitable cell (Figure 5G) in response to injecting square positive current pulses of varying amplitudes. Note that the excitable cell (Figure 5F) has much higher input resistance (a smaller current produced larger membrane potential change) compared to the non-excitable cells (Figure 5G).

### 3.6. Effects of Photothermal Response of Laminin-Coated AuNC-aECMs on the Expression of Voltage-Gated Na+ Channels in Differentiated Mature Neurons

**Figure 6:**
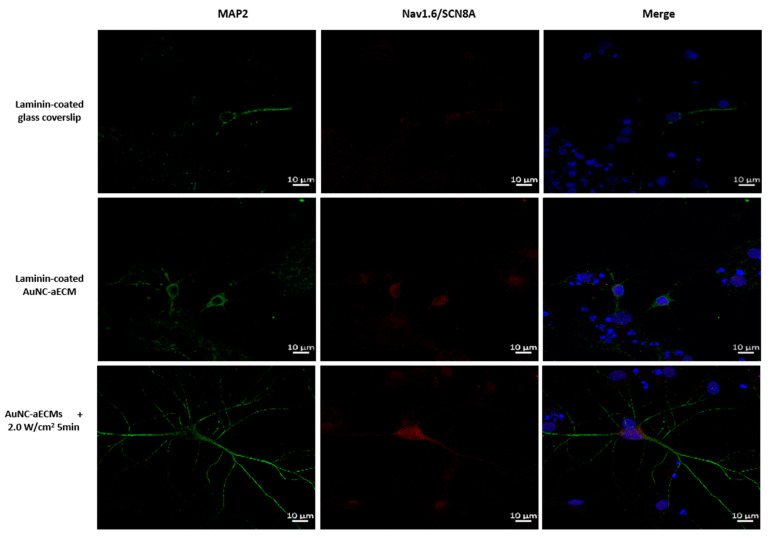
Immunofluorescence double staining of MAP2 and Na+ channels in differentiated neurons grown on laminin-coated AuNC-aECMs with/without laser irradiation. rNSCs were seeded onto both laminin-coated glass coverslips and laminin-coated AuNC-aECMs. After 2 days, the cells growing on laminin-coated AuNC-aECMs were treated with the laser irradiation parameter (2.0 W/cm^2^ for 5 min). After 14 days, cells were analyzed with immunostaining assay. Differentiated mature neurons and Na+ channels were identified with MAP2 (mature neuron marker) and Nav1.6 (Na+ channel marker), respectively.

Voltage-gated Na+ channels are widely distributed on the membranes of excitable cells such as neurons and are the most important ion channel required for neuronal cells to generate excitability and execute normal physiological functions [38,39,40,41]. Therefore, we investigated whether photothermal response of laminin-coated AuNC-aECMs increased the expression of voltage-gated Na+ channels (Nav1.6) compared to laminin-coated glass coverslips (Figure 6). As shown in Figure 6, compared with laminin-coated glass coverslips, the expression level of Nav1.6 channels was higher in both laminin-coated AuNC-aECMs with/without laser irradiation. Furthermore, increased Nav1.6 expression was observed in a laser intensity-dependent and laser exposure duration-dependent manner. Additionally, the colocalization of MAP2 (mature neuron marker) and Nav1.6 was observed in differentiated neurons grown on laminin-coated AuNC-aECMs with/without laser irradiation compared to laminin-coated glass coverslips.

### 3.7. Effects of Photothermal Response of Laminin-Coated AuNC-aECMs on the Expression of Heat Shock Protein 27, 70, and 90α in Rat Neural Stem Cells

**Figure 7:**
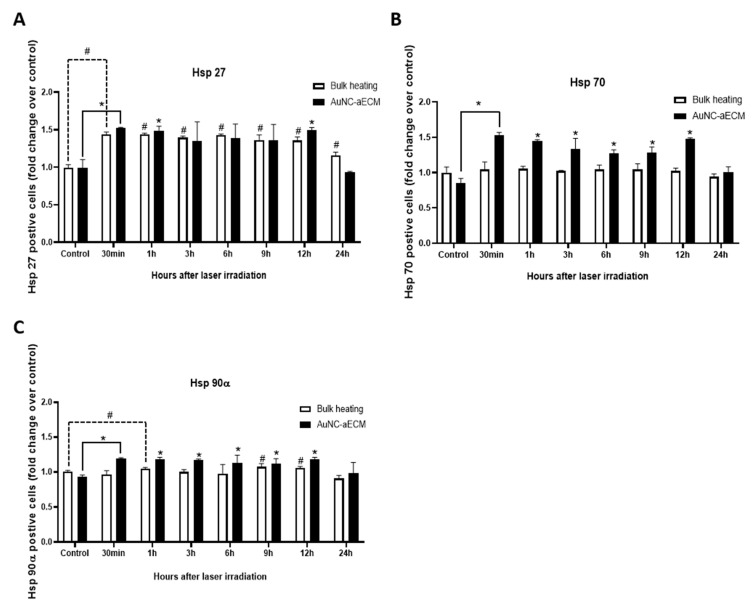
Assessment of heat shock protein 27, 70, and 90α expression in rNSCs. rNSCs were seeded onto laminin-coated AuNC-aECMs and after 4 days, the cells growing on laminin-coated AuNC-aECMs were treated with laser irradiation (2.0 W/cm^2^ for 1 min). The cells were collected at indicated time points and analyzed with flow cytometry to measure Hsp 27, 70, and 90α expression level (**A**–**C**). For bulk heating experiments, laminin-coated glass coverslips were placed into a 24-well plate, and rNSCs were seeded. After 4 days, plates were submerged in a circulating water bath at 43 °C for 30 min. The cells were collected with varying time points; then, the heat shock protein 27, 70, and 90α expression was measured by flow cytometry. Values represent the means ± SDs of three independent experiments. # *p* values < 0.05 vs. laminin-coated glass coverslip. * *p* < 0.05 vs. laminin-coated AuNC-aECM in the absence of laser irradiation group.

Hyperthermia promotes neuronal specification in NSCs in early stages of differentiation and increases neuronal differentiation by regulating the expression of heat shock proteins (Hsp) 27, 70, and 90α [42,43]. Therefore, we hypothesized that laser-induced thermal stimulation of laminin-coated AuNC-aECMs promotes the differentiation of NSCs into neurons and the development of immature neurons to maturity by regulating the expression of Hsp 27, 70, and 90α. To test this hypothesis, Hsp 27, 70, and 90α expression levels was measured post laser irradiation in rNSCs growing on laminin-coated AuNC-aECMs. As shown in Figure 7A, we found significantly increased Hsp 27 expression in laminin-coated AuNC-aECMs post laser irradiation at the early time points (30 min and 1 h) and 12 h compared to the control group. Furthermore, we found significantly increased Hsp 27 expression in all time points post hyperthermia induction using a bulk heating system compared to the control group. As shown in Figure 7B, we found significantly increased Hsp70 expression in laminin-coated AuNC-aECMs post laser irradiation from 30 min to 12 h. In contrast, there was no significant difference in Hsp 70 expression at bulk heating system. As shown in Figure 7C, we found significantly increased Hsp 90α expression laminin-coated AuNC-aECMs post laser irradiation from 30 min to 12 h compared to the control group. In addition, we found significantly increased Hsp 90 expression in 1, 9, and 12 h post hyperthermia induction compared to the control group in the bulk heating system. The data showed that the photothermal response of laminin-coated AuNC-aECMs significantly and predominantly increased Hsp 70 and 90α expression in rNSCs.

## 4. Discussion

A major challenge for developing a clinically suitable, human, neuronal cell source for regeneration of the damaged nerve tissues and neurons is efficient and controllable regulation of the differentiation of embryonic or induced pluripotent stem cell differentiation into desired cell types. Conventional approaches rely on the in vitro differentiation of NSCs into neurons through the expression of transcription factors [44,45,46], microRNA [47], and/or molecular signaling [48,49,50]. Furthermore, there are limited studies that attempt to maximize the differentiation of neural stem cells into neurons using the nanoscale topography of the matrix environment of the growth substrate. One group reported that a functionalized AuNRs nanocomposite system increased Schwann cell viability and was evaluated as a suitable substrate for the neural differentiation of human mesenchymal stem cells [51]. Another group reported that a thin grid pattern gold film coated with laminin resulted in only a 13.1% increase in neuronal differentiation compared to unpatterned substrates coated with laminin in neural stem cells [14]. However, complex processes of NSC differentiation result in poor neuron production and excessive glia cell differentiation, which occurs in an inefficient and/or uncontrollable manner.

To overcome these obstacles, we have found a means to improve the regulation of NSC differentiation processes by developing a new AuNC-aECM platform that increases the differentiation of NSCs into neurons.

In our previous study, we described the fabrication of a variety of types of nanoparticle-based aECMs by using gold nanorods, gold nanospheres, gold nanocubes, and silver nanospheres [31]. The ability to control the coverage density and aggregation of AuNPs onto silanized glass coverslips was achieved by using different types of reaction buffer, adjusting reaction buffer pH, salinity, temperature, duration of nanoparticle-functionalized glass reaction time, and silane concentration. We found that commercially obtained gold nanoparticles (gold nanorods, gold nanospheres, and gold nanocubes) showed significant responses to salinity and batch-to-batch variation of surfactant concentration. However, in-house synthesized AuNCs were used to overcome some of these limitations and yielded greater reproducibility and yield than commercially available materials. To increase potential for usage in a wide variety of biological applications, surfactants of nanoparticles could be replaced with proteins such as bovine serum albumin [52]. Furthermore, we demonstrated and modeled the highly localized photothermal effect of AuNC-aECMs by exposing them to a continuous wave laser. Our AuNCs as synthesized showed an extinction maximum at 755 nm, which shifted to 770 nm following attachment to the surface. As a result of the inherently broad nature of the LSPR peak, the extinction at 808 nm (laser wavelength) was greater than 90% of the maximum. Consequently, the purpose of the present study was to provide new information on how AuNC-aECMs and photothermal stress influences NSC proliferation, NSC differentiation into specific cell lineages, neuronal maturation, and to identify a potential molecular mechanism via chaperone (heat shock) protein expression. We demonstrated that the quantity of AuNCs deposited onto aECM surfaces can be finely controlled by our nanoparticle fabrication method (Figure 1A–D). Consequently, by optimizing the quantity of AuNCs in contact with a single cell, the magnitude and spatial distribution of membrane stimulation of adherent cells grown on laminin-coated AuNC-aECMs can be precisely controlled following laser irradiation (Figure 2A). The results suggest the hypothesis that lipid rafts, adhesion molecules, and other microdomains within the NSC plasma membrane physically interact with AuNCs deposited onto glass coverslips, which initiate remodeling of the microtubules and actin cytoskeleton toward the basal side of the NSC membrane that promotes NSC differentiation into neurons.

Previously, we studied how membrane stress influences the differentiation of NT2 cells into CNS neurons (hNT neurons) and other cell types. NT2 cells were exposed to extended trypsinization times (5–40 min) during the subculturing process and treated with varying electroporation parameters. We found that prolonged trypsin exposure (20 min) and a lower number of pulse electroporation (225V, 100 μsec pulse, 1.5 sec intervals, 2 pulses) increased neuronal differentiation and maturation in NT2 cells. In the current study and as shown in Figure 2B, we demonstrated that laminin-coated AuNC-aECMs increased the temperature of the surrounding medium in response to the applied laser intensity. The results implied that precise control of the amount of optical exposure delivered to laminin-coated AuNC-aECMs and produce photothermal responses that can be used as a stimulus for each cell. Therefore, we anticipated that the highly localized thermal stimulation induced by laminin-coated AuNC-aECMs post laser irradiation would induce membrane stress and further enhance NSC differentiation into neurons. In Figure 3, we showed that there was a slightly increased level of laser-induced neuronal differentiation on laminin-coated AuNC-aECMs post laser irradiation, although the difference was not significant. The results suggested that neuronal differentiation was maximized with laminin-coated AuNC-aECMs without laser irradiation, which is likely due to the effect of the nanoparticle-based ECM. Markedly, we found that the photothermal response of laminin-coated AuNC-aECMs exerted a beneficial effect in neuronal maturation by promoting neurite outgrowth. Figure 4 shows that laser-induced thermal stimulation of laminin-coated AuNC-aECMs increased the length of neuronal dendrites significantly in a laser intensity and duration-dependent manner. Noticeably, a wide range of dendrite length was observed at 2.0 W/cm^2^ for 1 and 5 min among laser irradiation groups. Specifically, one neurite is markedly longer than the other dendrites on the same neuron compared to differentiated neurons in other groups exhibiting symmetric dendrite lengths among all of their dendrites.

Astrocytes play important roles in NSC proliferation and the cell fate determination of NSCs [53,54], and astrocyte-conditioned medium has been added to NSC cultures in some studies to promote NSC proliferation and neurogenesis [55,56]. Noticeably, we observed that the differentiation of NSCs into astrocytes was diminished post laser-induced thermal stimulation of laminin-coated AuNC-aECMs as assessed by immunostaining. Astrocytes that were visualized in decreased populations had dwindled cytoskeletal structures. The results suggested that the photothermal response of laminin-coated AuNC-aECMs might down-regulate not only the NSC differentiation into astrocytes but also the astrocyte maturation processes. Consequently, this provided an optimal microenvironment for rNSCs to promote neuronal differentiation and maturation by securing a suitable space for rNSCs and possibly, secretomes secreted by astrocytes that play critical roles in promoting neuronal differentiation and maturation.

As shown in Figure 5A, cells growing on AuNC-aECMs exhibited spikelets of voltage in the absence of laser irradiation and after laser irradiation with 0.5 W/cm^2^ for 1 min, 2.0 W/cm^2^ for 1 min, and 2.0 W/cm^2^ for 5 min. However, we found no significant differences in the electrophysiological parameters among recorded cells. It is important to note here that the lack of detectable difference may not actually indicate that there was no effect of response to various treatments. This is because the recorded cells were quite diverse, and a large number of cells need to be recorded in each coverslip to account for the variability in electrophysiological properties. However, using the patch clamp technique, it was not possible to record more than a dozen cells per coverslip due to technical and time limitations (i.e., on average, one cell can be recorded each 15 min). In addition, we could identify an axon in 10 of the 20 cells that exhibited spikelets, suggesting that they are most likely due to differentiation of one of the neurites into an axon and the initiation of expression of voltage-gated Na+ channels near the axon hillock. As shown in Figure 6, we found an increased expression of voltage-gated Na+ channels in differentiated neurons grown on laminin-coated AuNC-aECMs post laser irradiation compared to the absence of laser irradiation. Additionally, we found increased colocalization of MAP2 and Nav1.6 in differentiated neurons grown on laminin-coated AuNC-aECMs in a laser intensity-dependent manner. The results suggested that immunocytochemistry might be more effective method to elucidate whether the photothermal response of laminin-coated AuNC-aECMs increases the differentiation of NSCs into functional neurons compared to the patch-clamping recordings.

As shown in Figure 5B–D, these data indicate that the majority of neurons exhibit extensive dendrites indicating an advanced stage of maturation, but only a few of those may later become functionally excitable neurons (i.e., exhibiting spikelets), and we speculate that this may occur via the trimming and reduction of dendrite number and promotion of a single axon. Therefore, longitudinal experiments are required to identify when differentiated neurons show initial signs of functionally excitable neurons and the underlying mechanisms of neuronal maturation processes.

As shown in Figure 7A–C, we found that the photothermal response of laminin-coated AuNC-aECMs specifically increased the expression of Hsp 70 and 90α and their expression level was higher than the bulk heating system, which suggests that the photothermal response of laminin-coated AuNC-aECM is more effective to induce NSC plasma membrane stress and regulate specific Hsps compared to canonical methods such as a bulk heating system. Further studies are needed to identify the signaling pathways by which the regulation of Hsps expression by the photothermal response of laminin-coated AuNC-aECMs plays a role in neuronal differentiation and maturation.

Our results suggest that there may be effective ways to utilize the unique features of nanomaterials combined with advanced cell culture approaches to generate supplies of specific CNS cell types such as functional neurons for further development. Ultimately, the use of laser irradiation and AuNC-aECMs to produce large quantities of functional neurons could be part of a protocol to transplant cells into damaged central nervous system tissues to provide replacement neurons and restore lost function. However, there are many factors to consider and optimize as this type of approach is expanded toward a specific goal for in vivo applications. Our control of the stress response by using photothermal-induced heating may allow for a non-invasive way to manipulate surfaces with progenitor cells that are inserted into the in vivo environment. Whether or not the substrate could be crafted out of a bio-friendly material and in a morphology that could be used in 3D tissue environments needs further exploration.

## Data Availability

Data sharing is not applicable to this article as no datasets were generated or analyzed during the current study.
